# Agglutinating mouse IgG3 compares favourably with IgMs in typing of the blood group B antigen: Functionality and stability studies

**DOI:** 10.1038/srep30938

**Published:** 2016-08-03

**Authors:** Tomasz Klaus, Monika Bzowska, Małgorzata Kulesza, Agnieszka Martyna Kabat, Małgorzata Jemioła-Rzemińska, Dominik Czaplicki, Krzysztof Makuch, Jarosław Jucha, Alicja Karabasz, Joanna Bereta

**Affiliations:** 1 Malopolska Centre of Biotechnology, Jagiellonian University in Kraków, Gronostajowa 7A, 30-387 Kraków, Poland; 2 Faculty of Biochemistry, Biophysics and Biotechnology, Jagiellonian University in Kraków, Gronostajowa 7, 30-387 Kraków, Poland

## Abstract

Mouse immunoglobulins M (IgMs) that recognize human blood group antigens induce haemagglutination and are used worldwide for diagnostic blood typing. Contrary to the current belief that IgGs are too small to simultaneously bind antigens on two different erythrocytes, we obtained agglutinating mouse IgG3 that recognized antigen B of the human ABO blood group system. Mouse IgG3 is an intriguing isotype that has the ability to form Fc-dependent oligomers. However, F(ab′)_2_ fragments of the IgG3 were sufficient to agglutinate type B red blood cells; therefore, IgG3-triggered agglutination did not require oligomerization. Molecular modelling indicated that mouse IgG3 has a larger range of Fab arms than other mouse IgG subclasses and that the unique properties of mouse IgG3 are likely due to the structure of its hinge region. With a focus on applications in diagnostics, we compared the stability of IgG3 and two IgMs in formulated blood typing reagents using an accelerated storage approach and differential scanning calorimetry. IgG3 was much more stable than IgMs. Interestingly, the rapid decrease in IgM activity was caused by aggregation of the molecules and a previously unknown posttranslational proteolytic processing of the μ heavy chain. Our data point to mouse IgG3 as a potent diagnostic tool.

Despite many years of research in the field of immunoglobulins, some aspects of antibody biology remain unexplained. There is an enormous volume of information concerning the structure and functions of human immunoglobulins, with these data mostly being collected during the invention of antibody-based therapeutics. In contrast, mouse immunoglobulins are less characterized, although their usefulness in research, diagnostics and the generation of genetically engineered chimeric and humanized therapeutics is undeniable^1^. Substantial differences between human and mouse antibodies are evident in the functions and properties of the IgG subclasses present in these two species^2^. Although similar in name, mouse and human IgG subclasses evolved after the divergence of the rodent and primate evolutionary lineages^3^,^4^; thus, the characteristics of human and mouse IgGs are not interchangeable.

Among four subclasses of mouse IgGs: IgG1, IgG2a, IgG2b, and IgG3, the least characterized is the IgG3 isotype. IgG3 has the lowest abundance among IgGs in mouse serum. Increased levels of IgG3 have been reported after immunization with polysaccharides^5^ and in autoimmune disorders, e.g., in the mouse model of systemic lupus erythaematosus^6^. In general, mouse IgG3s are able to interact with each other via Fc fragments in the absence of cognate antigens^7^,^8^. This interaction is very weak, with a K_D_ of approximately 10^−4^ M^9^, but its strength may be increased by binding certain antigens, especially multivalent antigens^10^. The exceptional ability of IgG3 molecules to form non-covalent oligomers is related to their cryoglobulin activity. Cryoglobulins are plasma proteins that precipitate reversibly at low temperatures^11^. The presence of cryoglobulins in serum is strongly correlated with autoimmune disorders^6^. In mouse models of autoimmunity, IgG3-containing immune complexes can induce renal disease and can even lead to death due to acute renal failure^12^,^13^. Strait *et al*.^14^ recently demonstrated that IgG1 may protect against the deposition of pathologic antigen-IgG3 antibody complexes in glomerular capillaries^14^.

Here, we investigated another unique property of mouse IgG3s that are specific towards antigen B from the human ABO blood group system. We discovered that the IgG3s obtained in our laboratory induced the agglutination of erythrocytes. This ability has previously been ascribed only to oligomeric IgMs, in which the distance between the two furthermost antigen-binding sites is 35 nm^15^. IgM induces haemagglutination very strongly and efficiently because it can easily bind antigens on two different erythrocytes and form cell aggregates. According to the current state of knowledge, IgGs cannot agglutinate red blood cell^16^. The span of the antigen-binding sites of IgG does not exceed 15 nm^17^ and is smaller than the minimum distance between two erythrocytes under physiological conditions. Therefore, it is believed that the IgGs are simply too small to induce haemagglutination.

Contrary to this generally accepted view, we proved that mouse IgG3 induce haemagglutination with similar efficacy as IgMs. The features of mouse IgG3 structure that are likely responsible for this ability were analysed. We also evaluated the applicability of IgG3-based reagents for blood typing and compared the stability of IgG3 and IgM in formulations that are used in serological diagnostics. The stability studies revealed a novel, protease-dependent, post-secretional processing of mouse IgM. In contrast, IgG3 was not subjected to this processing and might serve as a more stable serological reagent.

## Results

### Unique M18 antibody capable of red blood cell agglutination

**Figure 1:**
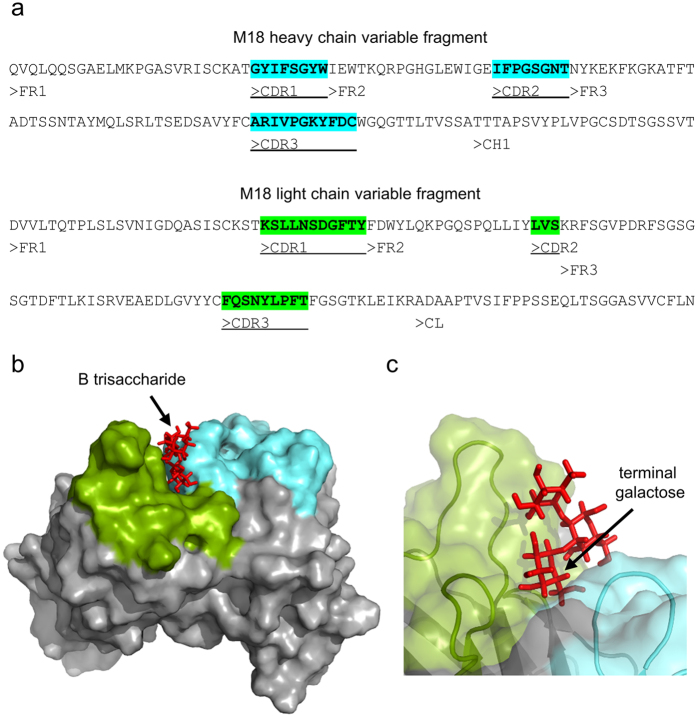
Variable region (V region) of M18 antibody. (**a**) V region sequences of M18. (**b**,**c**) Model of M18 V region complexed with its cognate antigen, blood group B trisaccharide (red). A molecular surface formed by CDRs is coloured using the same tints as in part a. The antigen was docked in a groove formed by the CDRs, where amino acid residues involved in the interaction with the antigen are most likely present. (**c**) Antigen in M18 binding site. The terminal galactose, unique for antigen B, is indicated.

We applied the hybridoma technique to generate biosimilar reagents suitable for serological diagnostics. The splenocytes of three mice immunized with group B red blood cells (RBCs) were used for four independent fusions. Media collected from cultures of all of the obtained hybridoma clones were screened for the presence of antibodies using slide agglutination tests with RBCs of group A, B and O. Antigens of the ABO blood group system are small, one-epitope oligosaccharides (Supplementary Figure 1)^18^. We obtained a total of 20 clones producing agglutinating anti-B antibodies (Table 1), and for all clones we sequenced cDNAs coding for variable regions. The analysis revealed that all of the specific clones generated by immortalization of splenocytes derived from a single RBCs-immunized mouse produced exactly the same antibody (Table 2). Therefore, by immunizing three mice, we obtained three different antibodies, M18, O10 and Q6, that were specific to the B antigen of the ABO blood group system. We expected all of the antibodies obtained to be of IgM class because only IgM is considered capable of RBCs agglutination in saline^16^. Indeed, two of the antibodies, O10 and Q6, were IgM κ and IgM λ, respectively. Unexpectedly, the third antibody, M18, was of the IgG3 κ isotype. Moreover, this IgG3 antibody appeared affinity-matured, displaying a high number of amino acid substitutions when compared to the germline sequence. Figure 1 presents a complete sequence of M18 variable (V) region and its molecular model. Both the amino acid sequence and the structure do not diverge from a typical mouse IgG3 molecule. The cell culture medium containing the M18 antibody induced a strong and rapid agglutination reaction, in contradiction with the agglutinating antibody paradigm. Thus, we decided to further investigate M18 and to compare its properties with IgMs that are currently used in serology.

### IgG3-induced haemagglutination depends on the IgG3 constant region

**Figure 2:**
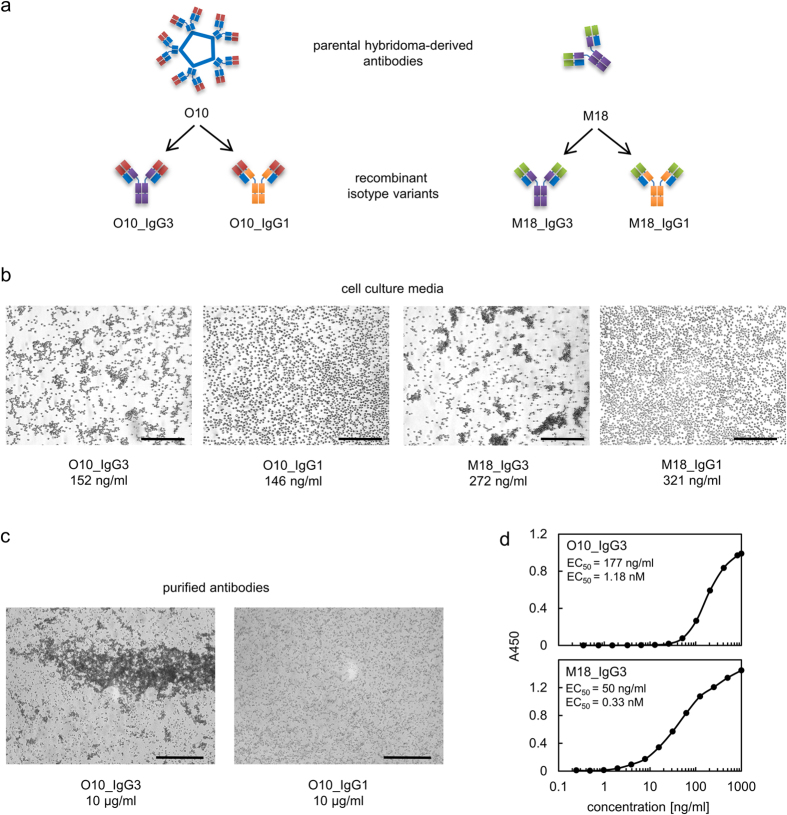
Haemagglutination induced by mouse IgG3. (**a**) The drawing schematically presents cloning of variable fragments into IgG3 and IgG1 frameworks. (**b**) IgG1 and IgG3 with the same variable fragments were compared in terms of their ability to agglutinate erythrocytes. Antibody concentration in the analysed cell culture media was determined using ELISA. Representative results of three independent experiments. Scale bar –100 μm. (**c**) Since O10_IgG3 was less efficient in haemagglutination than M18, the experiment presented in b was repeated using higher concentration of purified O10_IgG3 and O10_IgG1 antibodies. Representative results of two independent experiments. Scale bar –200 μm. (**d**) The EC_50_ of antibody binding to immobilized type B RBCs. Data points represent mean values obtained for triplicates of analysed samples. The experiment was performed two times. The EC_50_ of M18_IgG3 was approximately three times lower than the EC_50_ of O10_IgG3 in both repetitions.

As described above, we isolated an IgG3 capable of RBC agglutination. This ability may result from the structure of M18 V region, in which case it would be limited to this particular antibody, or from the structure of its constant region, in which case it would represent a general feature of the mouse IgG3 class. To investigate which part of the antibody was responsible for the agglutination property, we cloned the V regions of two agglutinating antibodies, M18 IgG3 and O10 IgM, into commercial mouse IgG1 and IgG3 frameworks (Fig. 2a). All of the newly obtained antibody variants retained the ability of parental molecules to bind the antigen (Supplementary Figure 2). Media collected from cultures of HEK293 cells expressing the antibodies or saline solutions of purified immunoglobulins were gently mixed with a 0.45% (haematocrit) suspension of group B erythrocytes for 20 min. Agglutination was evaluated using a microscope. M18_IgG3 and O10_IgG3 agglutinated erythrocytes, whereas the IgG1 switch variants of these antibodies did not induce haemagglutination (Fig. 2b,c).

Agglutination triggered by O10_IgG3 was weaker than that by M18_IgG3. As the EC_50_ of M18_IgG3 binding to immobilized type B RBCs was significantly lower than that of O10_IgG3, this difference was likely due to differences in the affinity of these antibodies towards the one-epitope B antigen (Fig. 2d). This effect is not surprising given that O10_IgG3 contained the variable fragment of the non-affinity-matured IgM, while M18 likely underwent affinity maturation (Table 2).

Overall, these results suggest that the ability to induce haemagglutination is a general feature of mouse IgG3s that recognize antigens present on erythrocytes.

### M18-induced agglutination is not dependent on the Fc fragment

**Figure 3:**
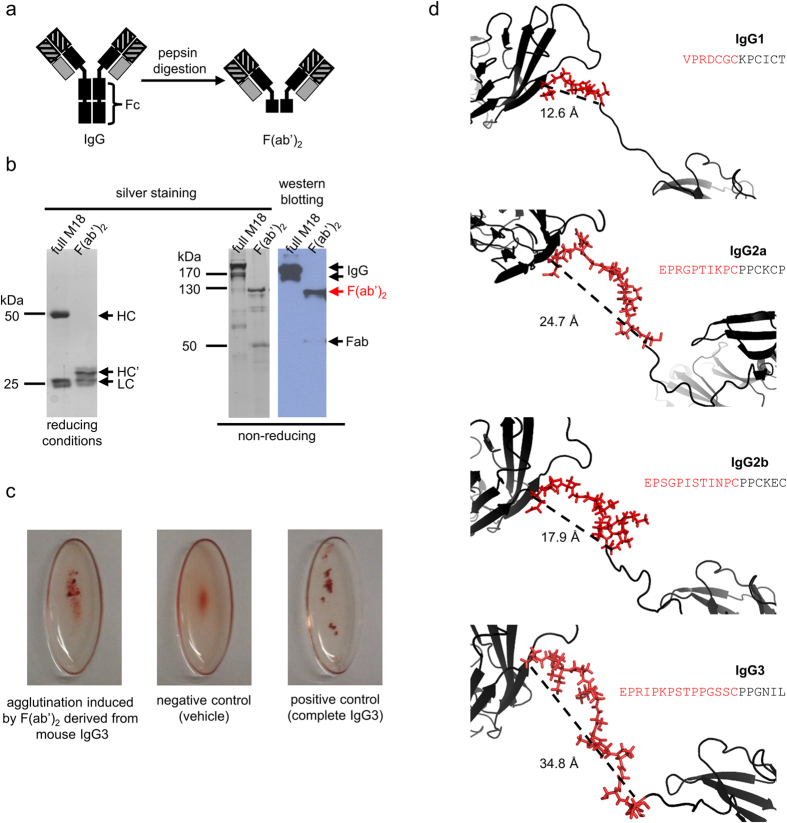
Structural determinants of mouse IgG3 agglutination capacity. (**a**–**c)** RBCs agglutination induced by F(ab′)_2_ of M18 antibody. (**a**) Pepsin digestion removes the Fc fragment from the IgG molecule, resulting in F(ab′)_2_. (**b**) Purity of M18 and its F(ab′)_2_. An intact IgG antibody under reducing conditions migrates as two bands corresponding to heavy chain (HC, approximately 50 kDa) and light chain (LC, 25 kDa). HC’ is a heavy chain digested with pepsin. F(ab′)_2_ is a protein with molecular mass of approximately 120 kDa, which was confirmed by the SDS-PAGE and western blotting under non-reducing conditions. Samples in western blotting were probed with anti-mouse-Ig antibody. IgG under non-reducing conditions migrates as two or three bands. A small amount of Fab is inevitably generated during pepsin digestion. (**c**) Haemagglutination of group B erythrocytes induced by mouse IgG3-derived F(ab′)_2_, vehicle buffer, and intact parental M18 antibody. (**b**,**c)** are representative results of five independent experiments. (**d**) Molecular modelling of the hinge region in M18 isotype variants. For each isotype a distance between alpha carbon of the first hinge amino acid and alpha carbon of the first cysteine residue involved in the inter-heavy chain disulphide bond is indicated. The sequence of the upper hinge region is presented for each subtype in red. For the sake of clarity, only one heavy chain was presented. The models of M18 IgG1, IgG2a and IgG3 variants were generated with C-score greater than 0.9, which indicates high confidence of the predicted models. C-score calculated for the M18 IgG2b model was slightly lower and reached 0.5 due to a lack of a complete mouse IgG2b structure in PDB database that could be used as a template in the modelling algorithm.

Mouse IgG3 antibodies have an ability, exceptional among murine IgGs, to weakly interact through their Fc fragment and to form oligomers upon antigen binding^6^,^9^,^19^,^20^,^21^. To analyse whether the Fc fragment is crucial for the agglutinating capability of IgG3, we removed the Fc portion of M18 via pepsin digestion. The enzymatic cleavage resulted in a highly pure divalent F(ab′)_2_ fragment of M18 (Fig. 3a,b). The obtained M18 antibody F(ab′)_2_ fragment retained the ability to agglutinate RBCs (Fig. 3c), indicating that the agglutination capability of M18 does not depend on its Fc fragment.

### Unique hinge region of IgG3 increases the span between antigen binding sites – in silico studies

As we excluded the role of the Fc fragment in the IgG3-induced agglutination, we focused on the F(ab′)_2_ fragment of the M18 antibody. The heavy chain of M18-derived F(ab′)_2_, which retains the ability to agglutinate, consists of the V region, the CH1 domain and a hinge region. We hypothesized that F(ab′)_2_ contains a motif, unique to IgG3, that spans the antigen-binding sites. To investigate if IgG3 diverges from other mouse IgG subclasses in this respect, we performed multiple sequence alignment of M18 IgG3 and M18 modelled as IgG1, IgG2a and IgG2b isotype variants (Supplementary Figure 3). The analysis revealed that the major differences between mouse IgG subclasses are in the hinge region. IgG3 has an extraordinarily long upper hinge, which is bordered by the first hinge amino acid and a cysteine, forming the first inter-heavy chain disulphide bond^22^. To analyse how differences in the upper hinge lengths between mouse IgG subtypes influence the theoretical range of antigen binding sites, we generated models of M18 isotype variants using comparative molecular modelling. The models indicated that the upper hinge of IgG3 is 1–2 nm longer than in other isotypes (Fig. 3d). Assuming that the Fab portion of an antibody has a constant size, the range of antigen binding sites should depend only on the upper hinge length. Based on two assumptions: (*i*) an immunoglobulin molecule has a planar conformation and (*ii*) the angle between the Fab fragments varies from 90° to 120°^23^, the *in silico* analysis indicates that the range of antigen binding sites in a divalent IgG3 molecule may be 2.8–3.4 nm longer than in other subtypes. This structural property likely explains the unique ability of IgG3 to agglutinate erythrocytes.

### Comparison of IgG3 and IgM as diagnostics reagents

We next compared the usefulness of IgG3 and IgMs in blood typing. Hybridoma cells were seeded at a density of 5 × 10^4^ cells/ml and cultured for 72 h. The antibody concentrations in media collected from the cultures and the corresponding antibody titres (as evaluated by slide agglutination; see Materials and Methods section: “RBCs and agglutination”) were as follows: 29 μg/ml and 64; 6 μg/ml and 16; and 15 μg/ml and 32 for clones producing M18 (IgG3), O10 (IgM) and Q6 (IgM), respectively. The specificity of the obtained antibodies was analysed using numerous freshly donated human blood samples of predetermined blood groups (Table 3). Each antibody was also tested in an indirect antiglobulin test and in agglutination of papain-treated RBCs. Negative results were consistently obtained when group A or group O blood samples were assayed. Importantly, M18, O10 and Q6 efficiently agglutinated RBCs from pregnant women and newborns (data not presented). Such RBCs may have reduced levels of ABO antigens^18^. To obtain antibody preparations with titres comparable to those of commercially available blood grouping reagents, we produced the antibodies using disposable bioreactors. The media collected from the cultures in bioreactors after cell separation and supplementation with buffers and preserving agents gave high-quality diagnostic reagents, which induced agglutination within 5–10 s and reached agglutination scores as high as 4+ within 1 min of contact between the reagent and a blood sample. Slide agglutination titres of M18-, O10- and Q6-based reagents were 256, 512 and 128, respectively.

We also compared the agglutination capacity of IgG3 and IgMs (Table 4). Three nanomolar solutions of M18, O10 and Q6 antibodies in saline were serially diluted and mixed with a 0.45% (haematocrit) suspension of RBCs. After 20 min of incubation, the results were evaluated using a phase-contrast microscope. We assumed that the IgMs were pentamers with a molecular mass of approximately 970 kDa. This assumption was made because: (*i*) the expression of J chain favours the secretion of pentameric IgMs^24^; and (*ii*) the analysed hybridomas, as well as Sp2/0 myeloma (fusion partner), expressed J chain (Supplementary Figure 4). With respect to IgG3 and IgM agglutination capacities, agglutination scores of 1+ were observed in the presence of 4–7 times more M18 molecules than for the two analysed IgMs (Table 4). The molecular mass of IgGs is approximately 6 times smaller than that of IgMs; thus similar amounts (in mass) of specific IgG3 and IgM are required to elicit the same degree of agglutination.

To verify whether the IgG3-based reagent may be more profitable than IgM-based reagents, we determined the productivity and doubling times of the clones that produced M18, O10 and Q6 antibodies. IgG3 was produced approximately three times more efficiently than IgMs (Supplementary Figure 5). The three analysed cell lines exhibited similar doubling times (Supplementary Figure 5); thus the overall antibody yields depend primarily on cell line productivity.

### Stability of IgG3- and IgM-based reagents

**Figure 4:**
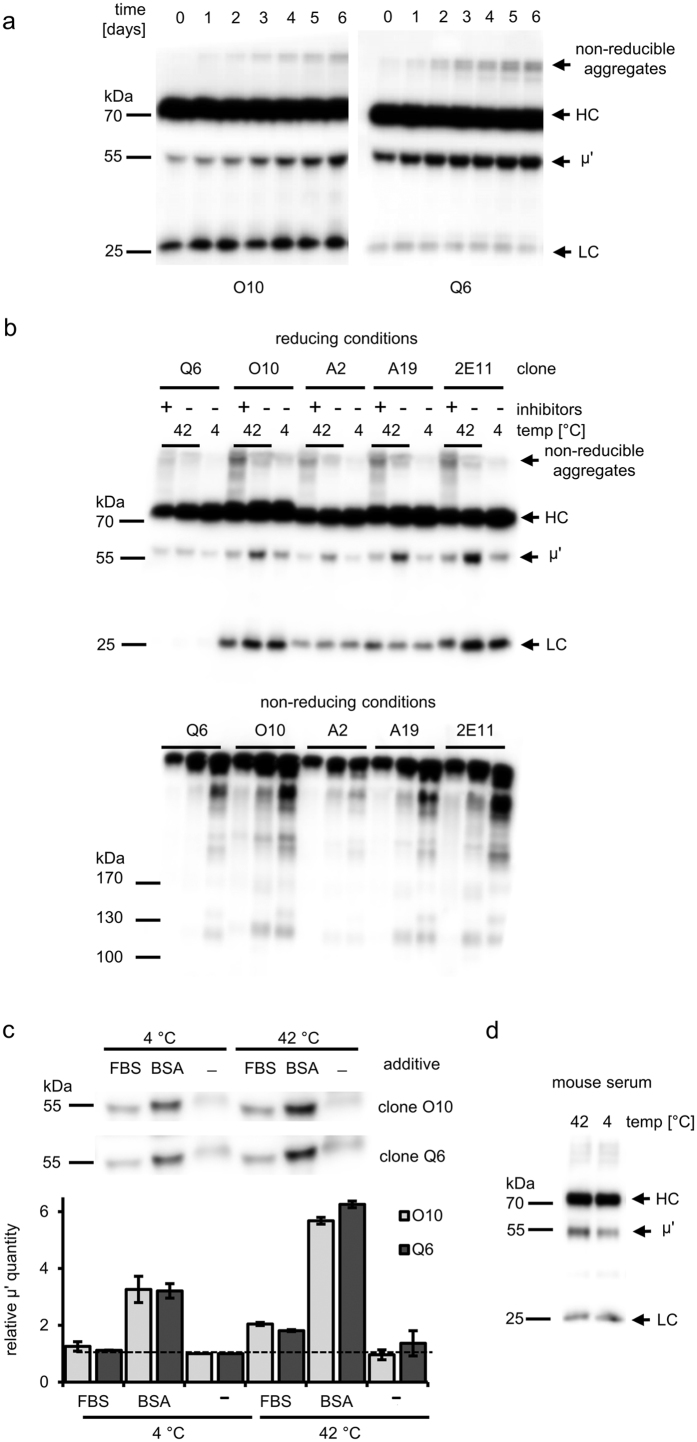
Chemical instability of IgM – western blotting analyses. All samples were probed with HRP-labelled anti-mouse IgM κ polyclonal antibodies. (**a**) O10- and Q6-based reagents were incubated at 42 °C for 6 days. Amount of truncated heavy chain (μ′) increased in subsequent samples taken daily. Images are representative of four independent experiments. (**b**) Protease inhibitor cocktail prevented trimming of a heavy chain, but promoted the formation of both reducible and non-reducible disulphide-based aggregates. Amount of IgM aggregates with the high molecular mass increased after exposure to elevated temperature. Representative results of four independent experiments are shown. Blots in (**b**) present the same samples resolved under reducing and non-reducing conditions. HC – heavy chain, LC – light chain. (**c**) Protease(s) present in serum trim(s) the IgM heavy chain. Purified IgM antibodies were incubated with 5% FBS or ht-BSA (1.5 mg/ml) for 4 days at indicated temperatures. The bar chart presents relative amounts of truncated heavy chain quantified using densitometry. The intensity of a band corresponding to the μ′ chain in the control sample without additive incubated at 4 °C was defined as 1. The μ′ chain in samples with additives migrates slightly faster than in control, probably due to the presence of the large band of serum albumin, which molecular mass is approximately 66 kDa. The chart presents mean values derived from two separate blots prepared from the same samples in one experiment. Results in (**c**) are representative of two independent experiments. (**d**) Truncation of μ chain occurs in mouse serum. Samples of mouse serum were incubated in parallel at 4 and 42 °C for 4 days. The figure presents a result obtained for one serum sample representative of three out of four analysed samples collected from different mice (in one sample the levels of μ′ were similar at 4 °C and 42 °C).

Monoclonal reagents used in serology, rather than being purified antibodies, are usually filtered and buffered media collected after fed-batch bioprocess. The reagents are often supplemented with preservatives and agents that enhance agglutination. To compare the stability of IgG3- and IgM-based reagents, we prepared simple formulations of M18, O10 and Q6 antibodies by buffering the culture media with 20 mM Tris-HCl pH 7.5 and adding 0.01% thiomersal. To accelerate any possible changes in their activity that may occur during storage, we used the classical procedure of keeping the formulations at 42 °C for 7 days^25^,^26^. The samples’ activities were tested daily via slide agglutination assays (Table 5). IgM antibodies lost their ability to agglutinate more rapidly than did IgG3. IgMs began to lose their functionality after 24–48 h at 42 °C. In contrast, IgG3 M18 was stable for at least 5 days at 42 °C. The results were consistent with our previous observation that IgMs, but not IgG3, relatively quickly lose their activity even when stored at room temperature. Moreover, O10 and Q6 could not be frozen and thawed without losing activity, in contrast to M18, which retained full activity even after 10 cycles of freeze-thawing (data not presented). Although the low stability of IgM is well known^27^,^28^, the molecular mechanisms responsible for the rapid loss of its activity remain unclear. Thus, we investigated the molecular basis of IgM instability. First, we used western blotting to analyse the samples that were collected during the accelerated storage study. As presented in Fig. 4a, during storage at 42 °C, (*i*) the μ heavy chain underwent truncation in the IgM-based reagents, resulting in a μ′ chain with a molecular mass of approximately 55 kDa; and (*ii*) non-reducible aggregates were formed in the IgM-based reagents. Both changes increased with time. To determine if the formation of truncated μ′ chain is a direct chemical or enzyme-mediated process, we incubated samples of five different IgMs, formulated as described above, with a protease inhibitor cocktail (Fig. 4b). The inhibitors prevented the increase in the levels of μ′ chains but promoted the formation of both reducible and non-reducible aggregates (Fig. 4b, lower panel). We next asked whether the proteases involved in μ chain trimming originated from hybridoma cells themselves or derived from the FBS. The latter was the case. Specifically, purified IgMs remained intact during storage at 4 °C or 42 °C but were trimmed in the presence of FBS (Fig. 4c). The truncation was even more pronounced when FBS was replaced with an equivalent amount of serum bovine albumin, which was obtained by heat treatment (ht-BSA). Although the reagent is supposed to contain primarily albumin, it is also enriched in numerous other proteins, as demonstrated in Supplementary Figure 6. The protease of interest most likely co-precipitates with albumin during the fractionation of the serum. The observed significant increase in IgM proteolysis upon replacement of FBS by BSA may also result from the removal of major plasma proteinase inhibitors, such as α-1-antiproteinase and α-2-macroglobulin during FBS fractionation. We also analysed whether the truncation of μ chain occurs in mouse serum. We observed an increase in μ′ chain levels after incubation of mouse serum samples at 42 °C for 4 days (Fig. 4e). This result indicates that the protease that trims the μ chain is present not only in bovine serum but also in species-compatible mouse serum and suggests that this mechanism of IgM instability is likely to occur *in vivo*.

To further characterize the differences in the stability of IgG3 (M18) and IgMs (O10 – home-made and MM30 – commercial) and to examine whether the storage of the formulations affects the stability of the immunoglobulins, we performed differential scanning calorimetric (DSC) analyses. DSC measures the enthalpy of protein unfolding that results from thermal denaturation. For small, one-domain proteins, thermograms that show a dependence of molar heat capacity of denaturation (c_p_) on temperature usually consist of a single heat absorption peak. The maximum of this peak, denoted T_m_, is the thermal transition midpoint and defined as the temperature where 50% of the protein is in its native conformation and the rest is denatured. The higher the T_m_, the more stable the protein. However, for multidomain proteins, such as immunoglobulins, the thermograms reflect a multi-state denaturation process and are more complex. This complexity originates from the merging of overlapping peaks that represent the thermal transitions of individual domains. The area under the curve, i.e., the integral of the molar heat capacity, equals the calorimetric enthalpy change (ΔH_cal_), which reflects the energy required for total protein unfolding.

**Figure 5:**
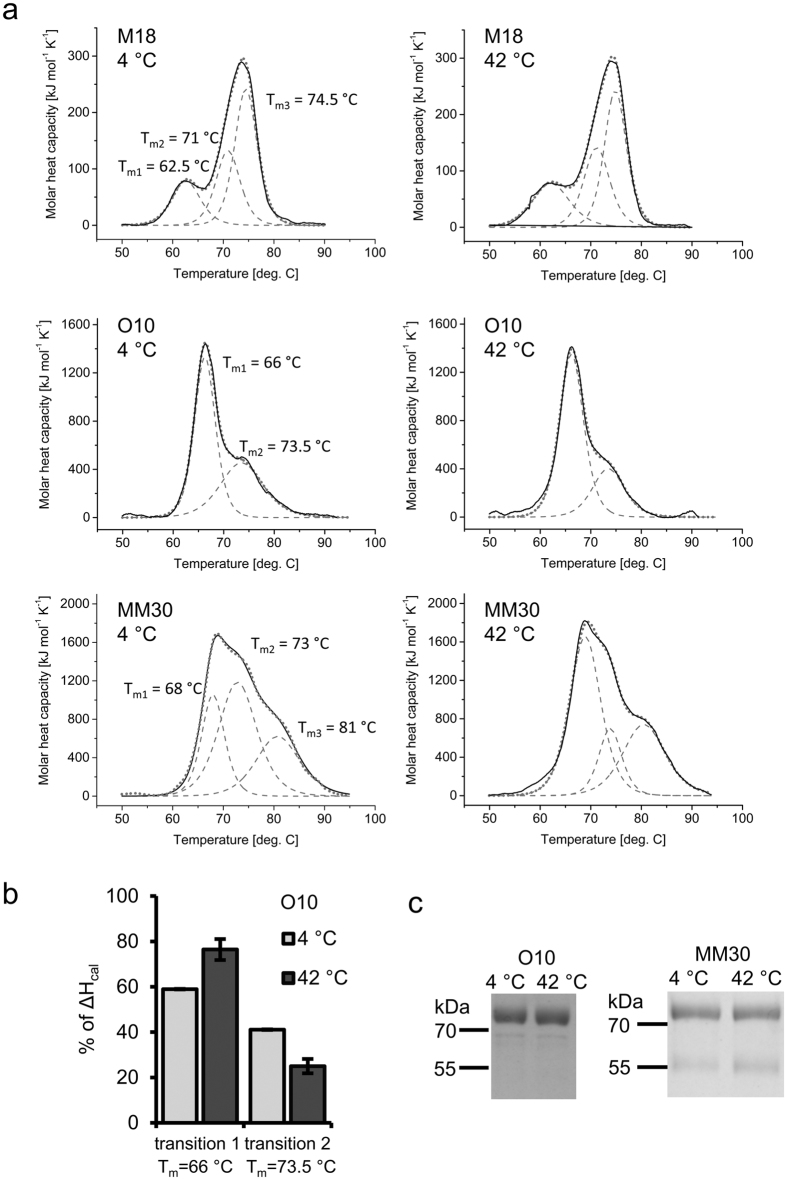
DSC analyses. (**a)** DSC thermograms of M18, O10 and MM30 samples stored at 4 °C as well as heated at 42 °C for 5 days. Black solid lines represent experimental data, which then were fitted with the two-state scaled model provided by TA Instruments (grey dashed lines). Dotted lines are sums of the fits. Maxima of the fits (T_m_) are indicated. Obtained T_m_ values were the same for both control and heated samples of the antibodies. (**b**) Contribution of the two separated transitions of O10 antibody to total ΔH_cal_. The chart presents mean values from two independent experiments. (**c**) SDS-PAGE of MM30 and O10 samples analysed with DSC. Proportion of truncated to full length heavy chain in MM30 samples was approximately 1:3 (densitometric analysis). The truncated heavy chain was almost undetectable in O10 samples.

Samples of the antibodies that were stored for 5 days at 4 °C (control) or 42 °C were subjected to DSC analyses (Fig. 5a). For each antibody, scans of control and heated samples resulted in the thermograms with a similar overall shape and ΔH_cal_. The maxima (T_m_) of the fitted two-state scaled transitions were the same for control and heated samples but differed for each antibody. The thermogram of M18 was deconvoluted into three overlapping peaks, which most likely corresponded to the CH2 (T_m1_ = 62.5 °C), CH3 (T_m2_ = 71 °C) and Fab (T_m3_ = 74.5 °C) transitions^29^. In the case of O10, the thermogram consisted of two transitions with T_m1_ = 66 °C and T_m2_ = 73.5 °C. The contributions of the first and second transition to total ΔH_cal_ observed for the control, non-heated O10, were 59% and 41%, respectively. The Fab fragments account for approximately 57% of the pentameric IgM mass (excluding N-glycans), whereas the remaining 43% is the mass of the Fc fragment. A consistency between the Fab:Fc mass proportion in a complete IgM and the relative contribution of the separate transitions in the O10 thermogram suggest that Fab domain unfolding precedes Fc fragment unfolding in this molecule. Although in the case of heated O10 the measured ΔH_cal_ was similar to the control samples, we observed a shift in the relative contributions of separate transitions (Fig. 5b). Scans of the MM30 samples showed broad thermograms, with at least three overlapping transitions. O10 and MM30 are both of the IgM class, but the analysed MM30 samples contained many more truncated heavy chains than the O10 preparations (Fig. 5c). We calculated the Fab:Fc mass proportion for MM30, taking into consideration the quantity of the truncated heavy chain. A direct comparison between the proportion and a contribution of the fits to ΔH_cal_ did not allow us to determine which transitions correspond to particular domains of the MM30 antibody.

In summary, the DSC analyses indicated that the stability of the M18 IgG3 Fab fragment is higher than that of O10 IgM. However, storage of either antibody for 5 days at 42 °C does not lead to evident structural changes that might be reflected by DSC profiles. Small changes in DSC profiles were observed for O10 IgM but not for M18 IgG3.

### Mouse immunization with RBCs leads to increased serum levels of IgM and IgG3

**Figure 6:**
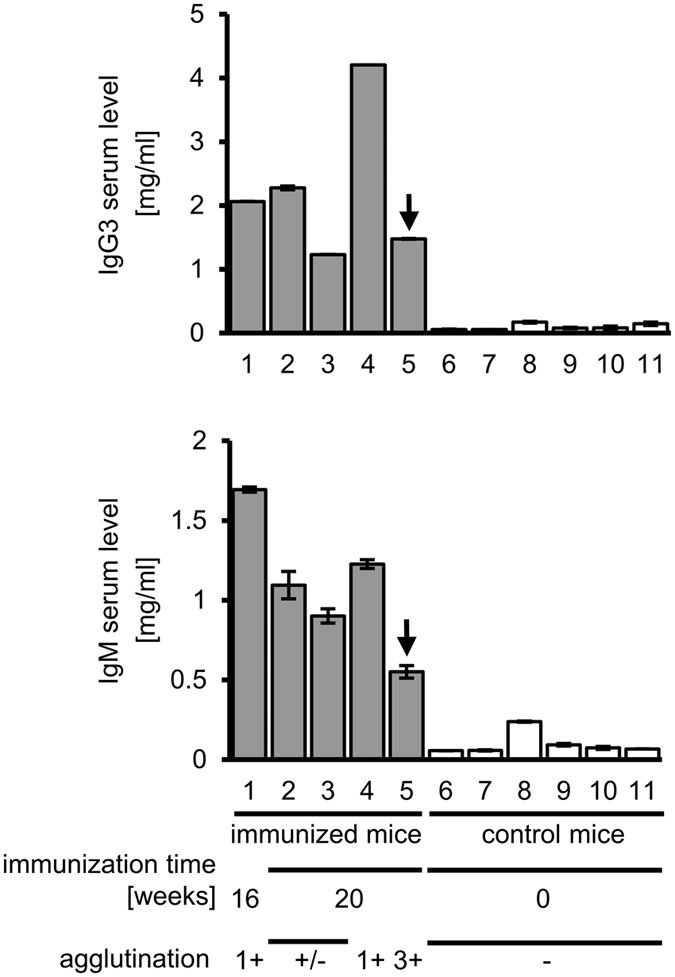
Serum IgG3 and IgM concentrations in RBCs-immunized mice. Samples of mouse serum were assayed using IgG3- and IgM-specific ELISAs. Samples from 11 different mice at age 25–29 weeks were analysed, among which 5 animals were immunized with RBCs. Arrows indicate serum sample of mouse no. 5, whose splenocytes were used in fusion resulting in M18 antibody generation. Agglutination was assessed for samples diluted 100 times. Error bars correspond to SD of concentration measurements.

To assess whether the mouse that was the splenocyte donor for the fusion that generated M18 had an exceptionally high IgG3 serum level, we compared concentrations of IgM and IgG3 in this mouse and in several other immunized and non-immunized mice (Fig. 6). As expected, all of the immunized mice had increased both IgM and IgG3 levels compared to controls; however, the serum levels of particular isotypes did not correlate with the isotype of the specific agglutinating antibody that was obtained (Table 2). Therefore, the generation of agglutinating IgG3 cannot be predicted on the basis of IgG3 and IgM concentration in the serum.

## Discussion

### Mouse IgG3 has a unique hinge region, which may underlie its agglutination capability

Atypical properties of mouse IgG3 have been reported since its discovery in 1971^8^. Among mouse IgGs, only IgG3 can interact (albeit weakly) via their Fc fragments. Although the estimated binding constant of the Fc-dependent interaction between mouse IgG3 molecules is low^9^, this self-association seems to have a tremendous impact on their properties^7^,^14^,^20^. The significance of the Fc fragment in mediating IgG3’s unique characteristics prompted us to determine whether this fragment is also responsible for the observed agglutination capability of IgG3. Unexpectedly, divalent F(ab′)_2_ prepared by pepsin-digestion of M18 retained agglutination capability, clearly indicating that M18-induced haemagglutination does not depend on the Fc fragment. We also prepared recombinant IgG1 and IgG3 isotype variants of two agglutinating antibodies, M18 (IgG3) and O10 (IgM). We showed that IgG3s with variable regions derived from M18 or O10 retained the ability to agglutinate RBCs, while corresponding IgG1 molecules did not induce direct haemagglutination. The results indicate that the ability to cross-link erythrocytes is a general feature of mouse IgG3, but not IgG1. To determine the molecular mechanism behind IgG3-triggered haemagglutination, we focused on the upper hinge of the antibody. This region determines the flexibility of an IgG molecule and indirectly influences its effector functions^22^. The upper hinge in mouse IgG3 is the longest of all mouse, rabbit and human IgG upper hinges. Moreover, the hinge region of mouse IgG3 is rigid, in contrast to the flexible hinges of other IgGs^22^.

*In silico* analysis was performed on model M18 isotype variants and indicated that the range of Fabs in IgG3 may be up to 3.4 nm longer than in other isotypes. However, the actual differences in the range of the Fab arms of different IgG subtypes are difficult to determine. Previously published reports concerning Fab ranges in mouse and human IgGs showed that the distance between Fabs depends on the antibody molecule geometry and its flexibility^17^,^23^. On the other hand, Sosnick *et al*. demonstrated that a functional Fab range does not directly correlate with the upper hinge length, e.g., mouse IgG1 and IgG2b have similar range of Fab arms, approximately 134 Å^17^, although the upper hinge of IgG2b is five amino acids longer than that of IgG1.

Comparative molecular modelling, which we applied in our work, does not provide a direct insight into the molecule dynamics but allows accurate measurements of theoretical distances between atoms. The *in silico* results suggest that mouse IgG3 has a uniquely extended span of Fab arms. We hypothesize that this feature of IgG3 accounts for its agglutination capability, and this hypothesis is supported by two experimental observations. First, all A- or B-specific antibodies that have been reported as suitable for serological diagnostics are of the large-span IgM class^30^,^31^,^32^; second, A- or B antigen-specific IgGs of an isotype other than IgG3 cannot agglutinate RBCs^33^,^34^. In summary, mouse IgG3 considerably differs from other IgG subtypes, especially in the extended length of the upper hinge. This characteristic may result in an unusually large range of its Fab arms and allow for the agglutination of RBCs.

### Proteolytic truncation of IgM heavy chain occurs extracellularly and depends on serum proteases

The co-expression of two IgM heavy chain isoforms, full-length (μ) and truncated (μ′), was observed several years ago^35^,^36^. The isoforms were detected in both human and mouse serum, as well as in supernatants from cell lines derived from these species. The truncated chain lacks a variable domain^35^,^36^. Previous studies have shown that the two isoforms are produced by translation of alternatively spliced transcripts of a single μ chain gene^35^. Here, we demonstrate that the μ′ chain can also be produced by proteolytic trimming of a full-length heavy chain. Thus, we provide evidence that IgMs may be subjected to posttranslational proteolytic processing that occurs extracellularly and is catalysed by an unidentified serum protease(s). We showed that these enzymes are present both in mouse and bovine serum. We are currently investigating the specificity of this putative enzyme(s), which may facilitate its identification.

Our findings have several important implications. IgMs are produced early during the adaptive immune response and can induce strong cytotoxic effects after binding to a cell-associated antigen. According to our results, a protease present in mouse serum cleaves off a variable fragment of the IgM heavy chain. The loss of this variable domains leads to reduced avidity and to the generation of trimmed molecules, the physiological roles of which are undetermined.

There are three Ig receptors that can bind IgMs: one that is exclusively IgM-specific (FcμR)^37^ and two that are IgM- and IgA-specific (pIgR and Fcα/μR)^38^. IgM binding to these receptors is independent of the antibody variable fragment^37^,^39^; thus, we speculate the following: (*i*) IgM transport across the epithelium should not be impaired by the truncated heavy chain, but trimmed molecules may provide inefficient immune protection; (*ii*) uptake of IgM-coated antigens by follicular dendritic cells and B-cells expressing Fcα/μR^38^ might be blocked by truncated IgMs; and (*iii*) the functions of FcμR-expressing B- and T-cells, as well as NK lymphocytes^38^,^40^, may also be modified by the trimmed IgM variants. Moreover, truncated IgMs likely have a reduced ability to activate complement. Our results suggest that the physiological functions of IgMs may be attenuated due to N-terminal trimming by serum proteases.

### IgM aggregation and μ heavy chain truncation lead to a rapid decrease in IgM activity

Research in the field of immunoglobulin structure, stability and formulation has been carried out for many years, and a vast amount of information concerning IgGs is available in the literature [reviewed by Wang *et al*.^26^]. In contrast to IgGs, factors and processes that influence the stability of IgMs are much less understood. Studies on IgM stability and formulations are important to pharmaceutical science because, due to their potent complement activation capabilities, IgMs are among the candidates for next generation antibody therapeutics^41^. In comparison to IgGs, IgMs are much less stable, and their production is demanding and inefficient^27^,^41^. Although the sensitivity of IgMs to elevated temperature and freezing is widely known, the exact molecular mechanism behind IgM instability is still not understood. In the present work, we used the accelerated storage test to investigate changes in the activity of IgM-based monoclonal reagents for blood typing. The analysed IgMs rapidly lost agglutination capability at 42 °C, an effect that was correlated with their aggregation and trimming of their heavy chains. Our results indicate that the observed aggregation is a dominant process that causes the loss of activity. According to western blotting analyses performed on samples that were resolved under reducing and non-reducing conditions, the aggregation seems to be primarily disulphide-based. However, significant levels of non-reducible aggregates were observed in IgM samples that were stored at 42 °C. The formation and exchange of disulphide bonds is the most common pathway that leads to IgG aggregation during storage^26^. We also report a specific and proteolytic trimming of the IgM heavy chain. The observed truncation occurred extracellularly and increased with time. We cannot determine how important the heavy chain truncation is for the IgM stability based on these results. We would like to emphasize that the activity of IgM-based reagents after 6 days of storage at 42 °C was significantly reduced or almost completely lost, despite there being a large amount of full-length heavy chain in the reagent. The attempts to prevent the trimming using protease inhibitors unexpectedly resulted in the promotion of aggregate formation, an effect that was likely induced by one or several active components of the inhibitor cocktail. Moreover, we hypothesize that the inhibition of heavy chain trimming itself may induce IgM aggregation by a currently unknown mechanism. One group has reported a decrease in the molecular mass of two mouse IgMs (from approximately 950 kDa to approximately 600–800 kDa) during storage in PBS over 180 days at 37 °C; this effect was accompanied by progressing aggregation. The aggregation and fragmentation were both clearly inhibited by lowering the pH to 5.5 and the addition of 20% sorbitol^28^. When all heavy chains in a pentameric IgM molecule were trimmed to 55 kDa polypeptides, the molecular mass of the whole oligomer decreased from 970 kDa to approximately 800 kDa, which is in the mass range reported in the cited work. Thus, it seems possible that the authors observed the heavy chain trimming catalysed by residual serum proteases. This trimming seems to be an initial step of IgM fragmentation and is followed by further proteolysis events, the loss of the light chain, and deglycosylation, resulting in a lowering of the IgM molecular mass to 600 kDa.

DSC analyses revealed that the Fab of M18 (IgG3) has a high T_m_, falling between 70 °C and 80 °C, which is the range for IgGs reported by other investigators^42^. The DSC thermogram of O10 (IgM) consisted of two transitions, the first of which likely originates from the Fab. Mueller *et al*.^28^ presented thermograms of eight different IgMs and, similar to our data, the highest peak (T_m_ between 63–68 °C at pH 7.5) appeared at the beginning of the thermal unfolding for each IgM^28^.

Comparison of the thermograms for IgGs and IgMs indicates that the denaturation of the Fab in IgM occurs at a lower temperature than for IgG. This effect may reflect differences in the stability of IgGs and IgMs that are stored at elevated temperatures. An unfolding event occurring at the lowest temperature during DSC scan is often considered as an indicator of the overall protein stability^43^. The first peak observed in IgG thermograms reflects the CH2 domain^29^. Although the CH2 unfolding has a lower T_m_ than the putative transition of the IgM Fab, it is reversible; thus, it should not affect the overall stability of the IgG^42^.

The influence of heating at 42 °C was noticeable in the case of O10 samples that were subjected to DSC experiments. The total ΔH_cal_ did not change after pre-treatment of O10 at elevated temperature. This result indicates that none of the domains of O10 stored at 42 °C for five days underwent significant unfolding. However, thermograms obtained for the control and preheated O10 samples differed in the relative contribution of to the total ΔH_cal_. We believe that this change reflects slight structural and/or chemical alterations that occurred during the accelerated storage of O10. The results that were obtained for MM30 suggest that the truncated heavy chain may influence the thermal unfolding of IgM, thus affecting the stability of the antibody during storage. To test this hypothesis, mutated IgMs that are resistant to truncation should be generated and their DSC profiles compared with those of parental proteins.

### Mouse IgG3s could replace IgMs in haemagglutination assays

Mouse IgG3s may be more feasible and interesting alternatives to IgMs, which are currently used in the serological determination of blood groups. IgMs have two major drawbacks in that (*i*) they are unstable, sensitive to heat and freezing; and (*ii*) their production and purification is usually inefficient and demanding^27^,^41^. As we have demonstrated, M18 IgG3 has an agglutination potential similar to IgMs but is advantageously durable and efficiently produced. Agglutinating IgG3s seem to be extremely rare. We were able to find only three reports of such antibodies. One of these, 2H12, recognizes glycophorin A^44^. In this case, the ability of IgG3 to agglutinate did not cause much surprise because glycophorin A protrudes significantly from the erythrocyte glycocalyx; thus, the distance between two epitopes on two cells is much smaller than the distance between cell surfaces. Another example is IgG3 clone 6.1.5, reported in 1984^34^ which is capable of the agglutination of both A- and B-group RBCs. However, this discovery was not translated into a diagnostic application due to broad specificity of the antibody. The third report, by Blanchard *et al*.^45^, concerned three IgG3s: NaM-1C9 (anti-A), NaM-1F6 (anti-A) and NaM-2E11 (anti-B). These IgG3s showed low haemagglutination potential, making them suitable for flow cytometry analysis of RBCs^45^.

In contrast to the previously reported IgG3 antibodies with similar specificity, M18 is an absolutely specific IgG3 antibody that exhibits a haemagglutination potential similar to IgMs. Thus, M18 may be used in routine blood typing based on direct haemagglutination testing.

On the basis of our results, we hypothesise that the ability of mouse IgG3 to agglutinate is an intrinsic trait of this molecule that results from its unique structure and does not depend on epitope localization. Although detailed studies on M18 structure-function relationship may provide a deeper insight into mouse IgG3 biochemistry, the validity of this hypothesis is still an open question. The generation of a genetically engineered mouse IgG3s with variable domains that are derived from an antibody that recognizes antigen D from the Rh blood group system could provide the answer. As the D antigen is localized very close to RBC plasma membrane^46^, obtaining anti-D agglutinating IgG3 would provide ideal evidence for our hypothesis that mouse IgG3s could replace all fragile IgMs in direct agglutination assays.

Overall, the M18 agglutinating antibody formulations compared favourably with IgM-based reagents and, given its major advantages in terms of stability and productivity, this antibody appears well suited for diagnostic applications.

## Materials and Methods

### RBCs and agglutination

Preserved standard human RBCs and samples of freshly donated blood were kind gifts from the Regional Centre of Blood Donation and Blood Treatment in Katowice, Poland. All of the blood samples tested negative for viral and bacterial pathogens. Agglutination tests, including indirect agglutination tests, were performed using the slide or tube technique according to the current World Health Organization guidelines^16^, unless otherwise stated. Agglutination was evaluated using a six-point scale, ranging from 4+, reflecting complete agglutination, through 3+, 2+, 1+, +/− and a negative score. The score ‘1+’ corresponds to weakly positive agglutination visible with the naked eye. Antibody titration was performed using a direct agglutination test with serial two-fold dilutions of the antibody preparation. The titre was the highest dilution that induced a 1+ agglutination score.

### Mouse immunization and hybridoma generation

Nine-week-old Balb/c mice, obtained from the Mossakowski Medical Research Centre, Polish Academy of Sciences, Warsaw, were immunized intraperitoneally with 2 × 10^7^ washed RBCs suspended in 150 μl of PBS. The cells were injected monthly, and at least 4 injections were given. Three days, two days and one day before the fusion, the mice were given a booster of an additional injection of RBCs. The hybridomas were generated using the protocol by Page and Thorpe^47^. The mouse myeloma cell line Sp2/0 was used as a fusion partner. Media collected from the wells with growing hybridoma clones were screened for antibody production using the slide agglutination test. The cells that produced antibodies that specifically and exclusively recognized antigen B from the ABO blood group system were propagated and further subcloned using the limited dilution technique to ensure monoclonality. All of the animal experiments were conducted in accordance with the standard guidelines by the National Ethics Committee on Animal Experimentation. The guidelines met the ethical standards required by Polish law (Animal Research Act, 2005) and by the European Directive 2010/63/EU on the protection of animals used for scientific purposes. All procedures involving mice were approved by the Animal Experiments I Local Ethics Committee, Kraków (Approval No. 71/2009) and all efforts were made to minimize animal suffering.

### Cell cultures and bioreactors

Hybridoma and HEK293 cells were cultured in DMEM with 4.5 g/l glucose (Lonza) and 5% FBS (Biowest) at 37 °C, 5% CO_2_ in a humidified incubator. To produce a formulation of antibodies at a high titre suitable for diagnostics, the hybridoma cells were adapted to BD Cell Mab Medium Quantum Yield medium (BD Bioscience) and cultured in two-compartment disposable CELLine1000 bioreactors (BD Bioscience). All of the cell cultures were routinely screened for *Mycoplasma spp*. contamination using PCR with rDNA-specific GPO1 and MGSO primers^48^.

### Analysis of nucleotide sequences of V regions

Genes encoding variable regions of antibodies were amplified as described by Wang *et al*.^49^ and sequenced using the dideoxy-chain termination technique (Genomed, Poland). The obtained sequences were analysed using the IMGT/V-QUEST program, version 3.3.5, provided by the International Immunogenetics Information System^50^,^51^.

### Isotype switching

cDNAs encoding the V fragments of the heavy and light chains were inserted into pFUSEss plasmids containing sequences that coded for the constant region of mouse γ1, γ3 or κ chain (Invivogen). The V fragment of a heavy chain was cloned into pFUSEss-CHIg-mG1 and pFUSEss-CHIg-mG3 vectors using *Eco*RI and *Afe*I restriction sites. Similarly, the V fragment of a light chain was inserted into pFUSE2ss-CLIg-mK plasmid using *Eco*RI and *Bst*API. All of the generated constructs were verified by sequencing (Genomed, Poland). The isotype variants were transiently expressed in HEK293 cells transfected using Lipofectamine 2000 (Invitrogen).

### Comparative molecular modelling

Comparative molecular modelling of immunoglobulins was performed on the I-TASSER server^52^ according to the protocol provided by the authors^53^. Models with the highest C-score, which indicates a high confidence of predicted models, were visualized and analysed using the PyMOL Molecular Graphics System, Version 1.3 (Schrödinger).

### Antigen docking

The M18 antibody structure obtained using comparative molecular modelling was simulated in GROMACS 4.6.5^54^,^55^ for 10 ns with a CHARMM36 force field under conditions similar to those published by Rahimi *et al*.^56^. The structure of the antigen, the blood group B trisaccharide, was obtained from PubChem, molecule ID: 10206531, and parametrized for CHARMM36 using CGenFF 3.01 automated topology builder^57^,^58^. Then, B trisaccharide was docked in the M18 model using Autodock Vina^59^. The structure of the antibody complexed with the docked antigen with the lowest binding energy was further simulated for 500 ps using the same parameters as before. Movement within the system was limited by a harmonic force of 10000 kJ × mol^−1^ × nm^−1^ between the protein and B trisaccharide, and position restraints were put on carbon atoms in the fucose ring.

### Antibody purification

CaptureSelect LC-kappa (mur) Affinity Matrix and CaptureSelect IgM Affinity Matrix (both from Thermo Fisher Scientific) were used for the purification of mouse monoclonal IgG3 and IgM antibodies, respectively. The purifications were performed according to the manufacturer’s instruction and were followed by dialysis against a buffer suitable for further procedures. The purity of antibodies was evaluated by SDS-PAGE and silver staining of the gels. The antibody concentration was determined using a BCA assay^60^, with bovine γ-globulin fraction (Pierce) as a reference.

### Fragmentation of IgG3 to F(ab′)_2_

Pepsin digestion of mouse IgG3 was performed essentially as described by Andrew^61^. The digestion time was experimentally optimized to 30 min. To separate F(ab′)_2_ fragments from residual full-length antibodies, the reaction mixture dialyzed against PBS pH 8.0 was loaded onto protein A chromatography column (Pierce), and the unbound fraction containing F(ab′)_2_, was collected.

### ELISA

A standard sandwich ELISA^62^ was used for measurements of antibody concentration. Mouse IgM B Cell ELISpot Development Module (R&D Systems) and a standard of mouse IgM (clone MM30, Abcam) were used for the quantification of IgMs. The concentration of IgG3 in mouse sera was determined using goat polyclonal capture antibody specific towards mouse IgG3 (Sigma) and a biotinylated goat anti-mouse kappa polyclonal detection antibody (AbD Serotec). Bound biotinylated antibodies were detected with HRP-conjugated streptavidin (R&D Systems). The concentrations of IgG3 and IgG1 in cell culture media after transient expression were evaluated using a goat polyclonal capture antibody that recognizes the mouse kappa chain (AbD Serotec), a rabbit monoclonal anti-mouse IgG1/IgG3 detection antibody (clone M111-2, Abcam), and a HRP-conjugated goat anti-rabbit polyclonal antibody. Purified mouse IgG3 (clone M18) and IgG1 (clone MCP21, Sigma) were used as standards. A colorimetric substrate for HRP OptEIA (BD Bioscience) was used in all experiments. The absorbance was read with a Molecular Devices Versa Max (Sunnyvale, USA) microplate spectrophotometer, and the concentrations were calculated using SoftMax Pro software.

### EC_50_ calculation

The EC_50_ was determined using ELISA on B-type RBCs, which were immobilized on the ELISA plates as described by Bigbee *et al*.^63^. Endogenous RBC peroxidase was blocked with 3% H_2_O_2_ for 20 min. The immobilized RBCs were incubated for 2 h with two-fold serial dilutions of HEK293 culture media containing transiently expressed M18_IgG3 and O10_IgG3 antibodies. The antibody concentrations in the media were determined using an IgG3-specific ELISA. The amounts of antibodies bound to RBCs were detected with biotinylated goat anti-mouse kappa polyclonal antibodies (1:3000, AbD Serotec), HRP-conjugated streptavidin (1:40000, Sigma) and a colorimetric substrate for HRP OptEIA (BD Bioscience). The obtained absorbance values were plotted against concentrations of the antibodies using an on-line tool ( www.ic50.tk), which was also used for the EC_50_ calculations.

### Accelerated storage studies

Purified antibodies in PBS or filtered cell culture media adjusted to pH 7.5 with 20 mM Tris-HCl were supplemented with 0.01% thiomersal (Sigma), a preservative commonly used for antibody formulations. M18, O10 and Q6 antibodies were in-house purified as described above. MM30 was purchased from Abcam. The media collected from cultures of O10, Q6, A19, A2 and 2E11 clones were used in the experiments. The in-house generated clones A19 and A2 produce IgMs that specifically agglutinate RBCs of blood group A, while the in-house generated clone 2E11 produces IgM specific towards a microbial antigen. The samples were stored at 42 °C and 4 °C for the indicated time periods. In some experiments, protease inhibitor cocktails (Sigma and Thermo Fisher Scientific) and 5 mM phenantroline (Bioshop) or appropriate volumes of their solvents (DMSO and methanol) were added to the culture medium. After the storage experiments, the samples were analysed using DSC or SDS-PAGE followed by western blotting.

### SDS-PAGE and western blotting

SDS-PAGE performed according to the standard protocol^64^ in 10% or 6% gels for reducing and non-reducing conditions, respectively. Protein separation was followed by a wet electrotransfer onto a PVDF membrane (Millipore)^65^. After blocking with 3% skim milk in PBS for 2 h, the membrane was incubated for 1 h with goat polyclonal HRP-labelled anti-mouse IgM kappa (AbD Serotec) or goat polyclonal HRP-labelled anti-mouse-Ig (BD Pharmingen) detection antibodies. The bands were visualized using Immobilon Western Chemiluminescent substrate for HRP (Merck Millipore) and analysed with a Fusion Fx apparatus with the Fusion Capt Advance Fx5 program (Vilbert Lourmat, France).

### Differential scanning calorimetry (DSC)

The DSC experiments were performed using NANO DSC Series III System with Platinum Capillary Cell (TA Instruments), with an active volume of 0.3 ml. To avoid bubble formation during heating mode, the samples were degassed prior to being loaded by pulling a vacuum of 30.4–50.7 kPa on the solution for a period of 10–15 min. The sample cell was then filled with 0.3 ml of sample solution, and an equal volume of buffer was used as a reference. The cells were sealed and thermally equilibrated for approximately 10 min at the starting temperature. All of the measurements were performed on samples under 0.304 MPa pressure. The data were collected in the range of 5–95 °C at a heating scan rate of 1 °C^−1^. Thermograms were corrected by subtraction of buffer blank scans and normalized to the protein concentration. Each data set was analysed for thermodynamic parameters with a software package supplied by TA Instruments. High values of the scaling factor (Aw) were allowed in the fitting algorithm.

## Additional Information

**How to cite this article**: Klaus, T. *et al*. Agglutinating mouse IgG3 compares favourably with IgMs in typing of the blood group B antigen: Functionality and stability studies. *Sci. Rep.* **6**, 30938; doi: 10.1038/srep30938 (2016).

## Supplementary Material

Supplementary Information

## Figures and Tables

**Table 1:** Summary of fusions.

Fusion^a^	Clones tested	Clones producing agglutinating mAbs		Name of the specific antibody used in further experiments
		non-specific	specific	
M	2625	6	**3**	M18
O	1784	1	**6**	O10
Q, U	2854	14	**11**	Q6

^a^Letters denominate independent fusions; Q, U – splenocytes from one mouse were used for two fusions.

**Table 2:** Analysis of specific antibody sequences.

Fusion	Genes coding for a variable fragment of the specific mAb^a^ (identity with the closest V-region)		Mutations^b^ HC/LC	Amino acid substitutions^c^ HC/LC	Isotype
	Heavy chain (HC)	Light chain (LC)			
M	IGHV1-9*01 F (94%)IGHJ2*01 F (94%)IGHD2-4*01 F	IGKV1-99*01 F (95%)IGKJ4*01 F (100%)	16/6	12/3	IgG3 κ
O	IGHV6-7*02 F (96%)IGHJ4*01 F (96%)IGHD1-1*02 F	IGKV1-110*01 F (96%)IGKJ1*01 F (100%)	5/2	3/2	IgM κ
Q, U	IGHV4-1*02 F (97%)IGHJ3*01 F (92%)IGHD5-8*01	nd^d^	3/nd	0/nd	IgM λ

^a^Sequence analysis revealed that all specific clones derived from a single mouse produce exactly the same mAb.

^b^Mutations with respect to the germline sequence were analysed within FR1-FR3.

^c^Amino acid substitutions with respect to the germline sequence were analysed within FR1-FR3.

^d^Not determined. Despite many efforts we did not manage to design λ-specific primers allowing for selective amplification of a productive assemblage of λ VJ-genes.

**Table 3:** Specificity of M18, O10 and Q6 antibody evaluated using agglutination of fresh blood samples.

blood group	M18		O10		Q6	
	donations	positive results	donations	positive results	donations	positive results
0	20	—	20	—	13	—
A_1_	17	—	5	—	8	—
A_2_	13	—	5	—	3	—
A_1_B	17	17	2	2	3	3
A_2_B	6	6	2	2	—	—
B	52	52	30	30	13	13

**Table 4:** Comparison of IgM and IgG3 agglutination capacity.

concentration [nM]	O10 (IgM) samples			Q6 (IgM) samples			M18 (IgG3) samples		
	I^a^	II	III	I	II	III	I	II	III
3.00	2+	2+	2+	3+	3+	3+	1+	2+	2+
1.50	2+	2+	1+	2+	2+	3+	+/−	1+	1+
0.75	1+	1+	+/−	1+	1+	1+	—	—	—
0.38	1+	+/−	+/−	1+	1+	1+	—	—	—
0.19	+/−	+/−	+/−	1+	1+	1+	—	—	—
0.09	—	—	—	+/−	+/−	+/−	—	—	—
0.05	—	—	—	+/−	—	—	—	—	—
0	—	—	—	—	—	—	—	—	—

^a^Presented results are from three independent experiments designated as I, II and III.

**Table 5:** Stability of IgM and IgG3 antibodies at 42 °C.

Time [days]	O10 (IgM)		Q6 (IgM)		M18 (IgG3)	
	duplicates		duplicates		duplicates	
	I	II	I	II	I	II
0	16^a^,^b^	32	32	32	32	32
1	16	16	16	16	32	32
2	16	8	4	8	32	32
3	8	8	—	2	32	32
4	8	8	—	2	32	32
5	8	8	—	2	32	32
6	4	8	—	—	16	32
7	2	4	—	—	16	16

^a^Agglutination-inducing titres of the antibodies after indicated time of heating.

^b^Representative results of three independent experiments performed in duplicates are shown.
