# Supplementary material for: Agglutinating mouse IgG3 compares favourably with IgMs in typing of the blood group B antigen: Functionality and stability studies
Source: Sci Rep. 2016 Aug 3;6:30938. doi: 10.1038/srep30938 (PMC4971511; doi:10.1038/srep30938)
Supplement: Supplementary Information [file srep30938-s1.pdf]

Agglutinating mouse IgG3 compares favourably with IgMs in typing of the blood group B antigen: Functionality and stability studies.

Authors:

Tomasz Klaus[1,2], Monika Bzowska[1,2], Małgorzata Kulesza[2], Agnieszka Martyna Kabat[2], Małgorzata Jemioła-Rzemińska[1,2], Dominik Czaplicki[2], Krzysztof Makuch[2], Jarosław Jucha[2], Alicja Karabasz[2], Joanna Bereta[2]*

[1] Malopolska Centre of Biotechnology, Jagiellonian University in Kraków, Gronostajowa 7A, 30-387 Kraków, Poland

[2] Faculty of Biochemistry, Biophysics and Biotechnology, Jagiellonian University in Kraków, Gronostajowa 7, 30-387 Kraków, Poland

*Corresponding author: Joanna Bereta, phone number: +48 12 664 63 56, fax number: +48 12 664 69 02, e-mail address: joanna.bereta@uj.edu.pl

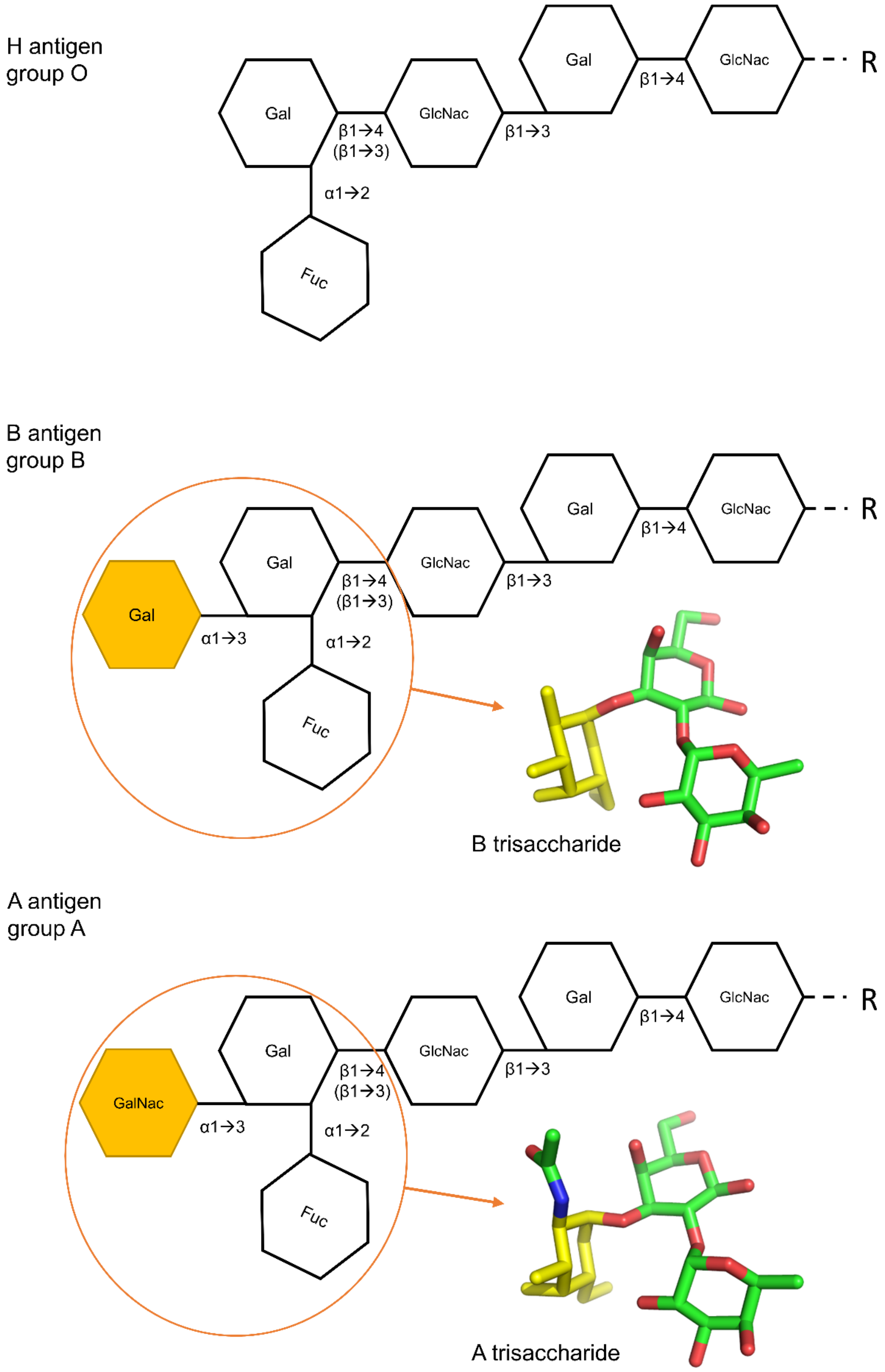

H antigen
group O
Gal
GlcNac
Gal
GlcNac
R
β1→4
(β1→3)
β1→3
β1→4
α1→2
Fuc
B antigen
group B
Gal
Gal
GlcNac
Gal
GlcNac
R
α1→3
β1→4
(β1→3)
β1→3
β1→4
α1→2
Fuc
B trisaccharide
A antigen
group A
GalNac
Gal
GlcNac
Gal
GlcNac
R
α1→3
β1→4
(β1→3)
β1→3
β1→4
α1→2
Fuc
A trisaccharide

Supplementary Figure 1 – **Antigens of the ABO blood group system.** A and B trisaccharides, which are commonly used in structural studies [1-3], are indicated with orange circles. A antigen differs from B antigen only by one substituent at C2 of the terminal galactose.

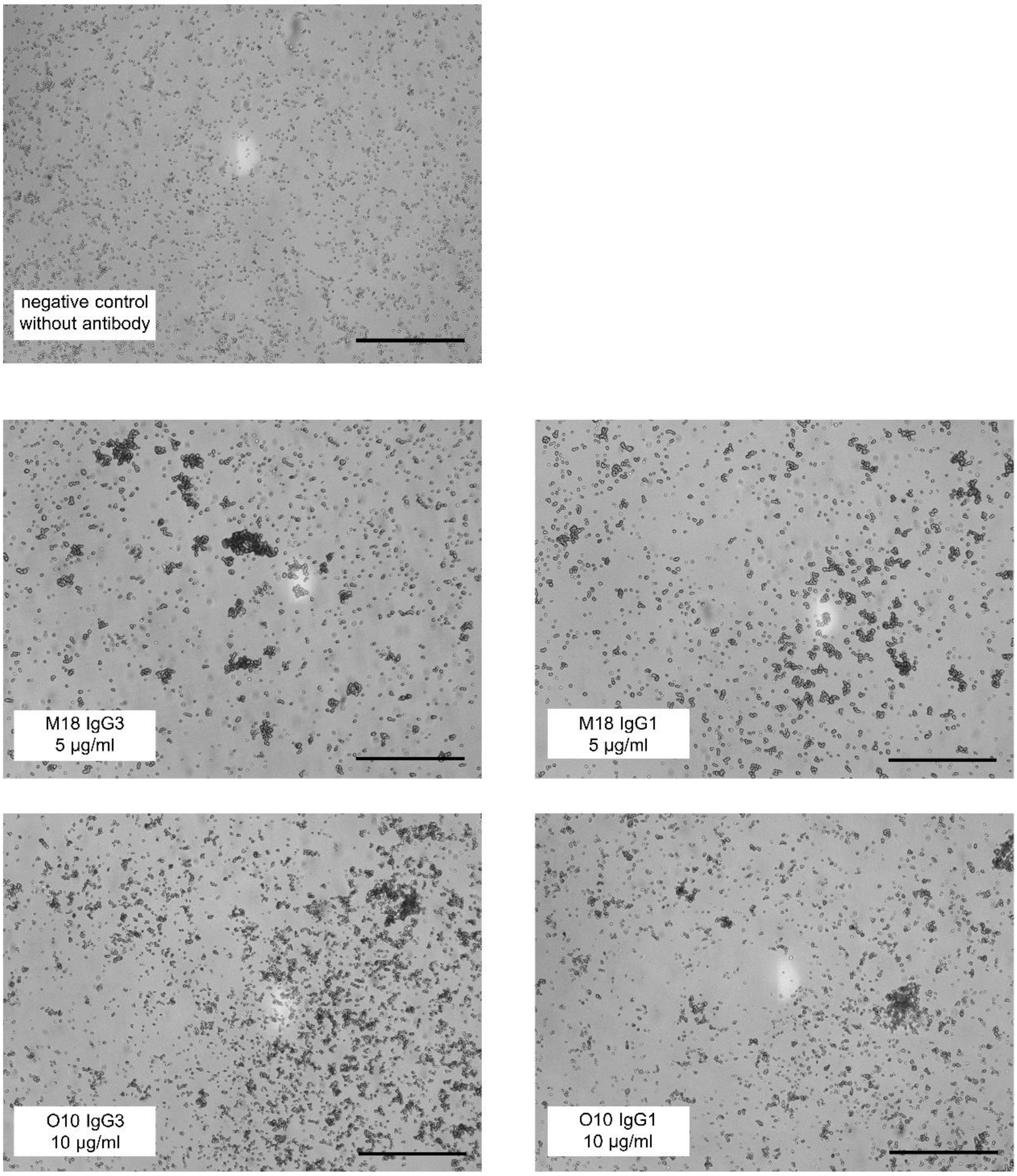

Supplementary Figure 2 – **IgG3- and IgG1-triggered agglutination of papain-treated erythrocytes.** Purified isotype variants of O10 and M18 were gently mixed with 0.45% (haematocrit) suspension of papain-treated group B erythrocytes for 20 min. The agglutination was evaluated using a microscope. The papain treatment reduces the negative charge of the erythrocyte surface. Thus, papain-treated erythrocytes can be agglutinated by IgGs. All generated recombinant IgG antibodies agglutinated group B erythrocytes, which proved that they retained the ability to bind the antigen after isotype switching. Representative results of two independent experiments. Scale bar – 200 µm.

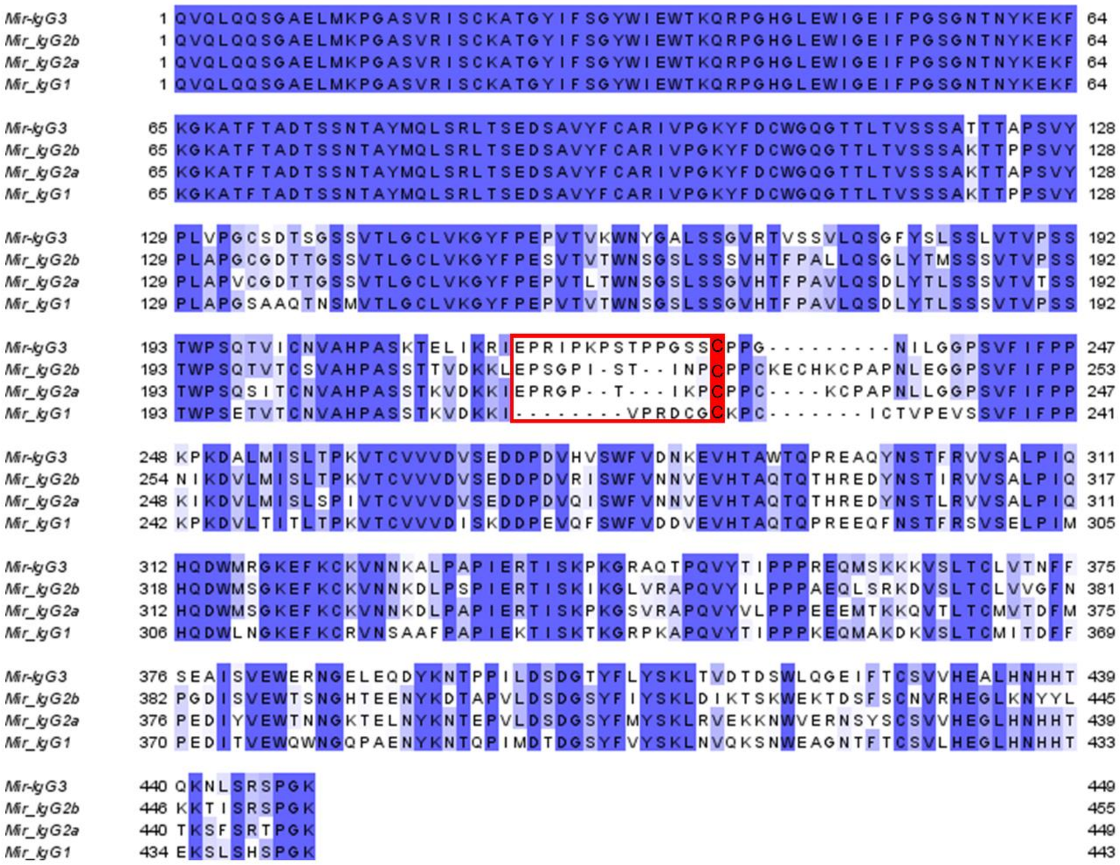

Supplementary Figure 3 – **Multisequence alignment of M18 isotype variants**. The alignment was done using Clustal-omega 1.1.0 [4] and manually corrected according to the previously published alignment [5]. Sequences are colored by conservation. The red frame indicates the upper hinge region. The cysteine residues involved in the first disulfide bond between heavy chains are highlighted in red.

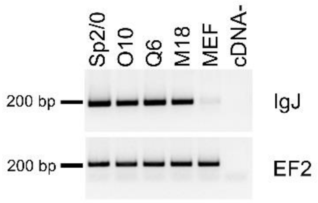

Supplementary Figure 4 – **Expression of J chain in Sp2/0 and hybridoma cell lines producing O10, Q6 and M18 antibodies.** RNA was isolated from the cells and transcribed into cDNA using M-MLV reverse transcriptase (Promega) according to the manufacturers' instruction. Then PCR was performed with primers specific to IgJ transcript and *EF2*, a housekeeping gene. MEF – mouse embryonic fibroblasts, negative control. Representative result of two independent experiments.

IgJ specific primers: for TGTAACAGGTGACGACGAAGC

rev GGGGAGGTGGGATCAGAGATA

EF2 specific primers: for GCGGTCAGCACAATGGCATA

rev GACATCACCAAGGGTGTGCAG

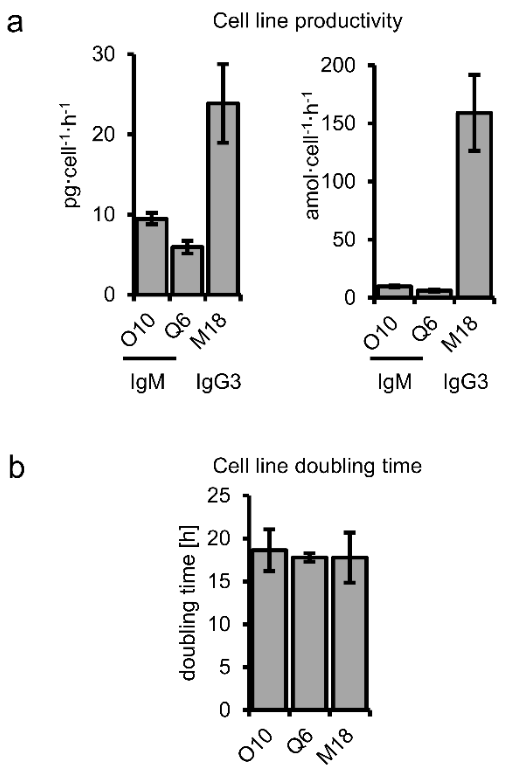

Supplementary Figure 5 – **IgMs and IgG3 production efficiency. a** – Cell line productivity presented in two different units. Molecular masses of IgM and IgG3 are 970 kDa and 150 kDa, respectively. **b** – Cell line doubling time calculated for the logarithmic growth phase. Bars in **a** and **b** present mean values from three and two independent experiments, respectively. Error bars correspond to SD.

Determination of cell line doubling time

The doubling time of each cell line was calculated according to the protocol developed by Murhammer [6].

Analysis of cell line productivity

Hybridoma cells in mid-logarithmic growth phase were seeded at a density of 2.5×10^4 cells/ml and cultured for 72 h. Then the cultures were harvested, the cells were counted using a hemocytometer and their viability was determined using trypan blue exclusion. The

antibody concentrations in the collected culture media were measured using ELISA. To calculate the cell line productivity we made two assumptions, consistent with our experimental observations: (*i*) cells constantly produce similar amounts of antibody, and (*ii*) cells divide at a steady rate. The number of cells L in time t after the culture inoculation is expressed by the formula: $L = A \times 2^{\frac{t}{\tau}}$, where A refers to the initial cell number and τ is the doubling time. An integral of the function $\int A \times 2^{\frac{t}{\tau}}\,dt = \frac{A}{ln2} \times 2^{\frac{t}{\tau}}$ equals the cumulative cell lifespan expressed in [cell×h] unit. To calculate the cell line productivity, the amount of antibody was divided by the above-described integral. The productivity was expressed as a quantity (pg or amol) of antibody produced by a single cell per time unit (h).

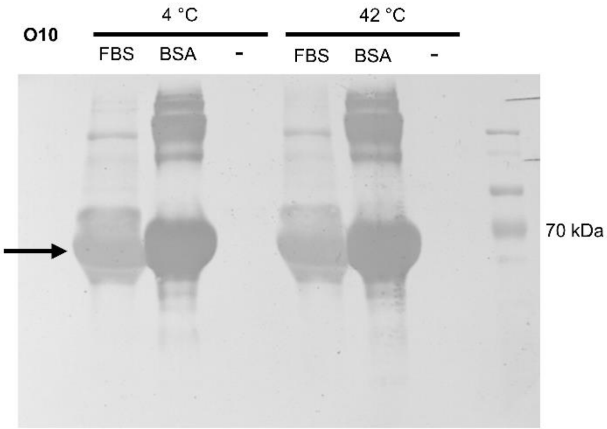

Supplementary Figure 6 – **Comparison between sets of proteins present in FBS and a ht-BSA sample**. Coomassie Brilliant blue staining of the blot used for the western blotting analysis of IgM fragmentation (upper panel of Fig. 4c in the main article). The arrow indicates the band corresponding to serum albumin.

1 Bu, W. *et al.* Structural basis for the receptor binding specificity of Norwalk virus. *J Virol* **82**, 5340-5347, doi:10.1128/JVI.00135-08 (2008).

2 Cao, S. *et al.* Structural basis for the recognition of blood group trisaccharides by norovirus. *J Virol* **81**, 5949-5957, doi:10.1128/JVI.00219-07 (2007).

3 Higgins, M. A. *et al.* Differential recognition and hydrolysis of host carbohydrate antigens by Streptococcus pneumoniae family 98 glycoside hydrolases. *J Biol Chem* **284**, 26161-26173, doi:10.1074/jbc.M109.024067 (2009).

4 Sievers, F. *et al.* Fast, scalable generation of high-quality protein multiple sequence alignments using Clustal Omega. *Mol Syst Biol* **7**, 539, doi:10.1038/msb.2011.75 (2011).

5 Dangl, J. L. *et al.* Segmental flexibility and complement fixation of genetically engineered chimeric human, rabbit and mouse antibodies. *Embo J* **7**, 1989-1994 (1988).

6 Murhammer, D. W. Useful tips, widely used techniques, and quantifying cell metabolic behavior. *Methods Mol Biol* **388**, 3-22, doi:10.1007/978-1-59745-457-5_1 (2007).
